# Listeria monocytogenes Infective Endocarditis With Concurrent Bacterial Meningitis and Systemic Embolization: A Case Report and Literature Review

**DOI:** 10.7759/cureus.97708

**Published:** 2025-11-24

**Authors:** Aoumar G Chamma, Wendy Saliba, Linda Chamma

**Affiliations:** 1 Cardiology, University of Balamand, Beirut, LBN; 2 Urology, Lebanese University, Beirut, LBN

**Keywords:** bacterial meningitis, cerebral septic emboli, hepatic lesions, infective endocarditis, listeria monocytogens

## Abstract

Listeria monocytogenes is an uncommon cause of infective endocarditis (IE), and the concurrent occurrence of Listeria IE with bacterial meningitis and systemic septic embolization is exceedingly rare.

We report the case of a 74-year-old man who presented with fever and altered mental status and was found to have L. monocytogenes IE complicated by concurrent meningitis and multiple septic emboli, including cerebral infarcts, a splenic infarct, and hepatic microabscesses. Blood and cerebrospinal fluid cultures grew L. monocytogenes, confirming the diagnosis. He was treated with high-dose intravenous ampicillin plus gentamicin, as L. monocytogenes is intrinsically resistant to cephalosporins and the combination of an aminopenicillin with an aminoglycoside is the treatment of choice. The patient improved with therapy and completed a six-week course of antibiotics, achieving a favorable outcome.

This case highlights the importance of rapid diagnosis and timely initiation of targeted antimicrobial therapy, as early treatment with ampicillin and gentamicin remains crucial for patient recovery and for reducing the high mortality associated with this rare but life-threatening infection.

## Introduction

Listeria monocytogenes is a gram-positive facultative intracellular bacillus. It functions as a foodborne pathogen that leads to gastroenteritis, bacteremia, and central nervous system (CNS) infections, including meningitis and encephalitis [1-4]. Invasive listeriosis predominantly affects the elderly, immunosuppressed individuals, pregnant women, and neonates [3,4]. The occurrence of L. monocytogenes infective endocarditis (IE) represents a rare but dangerous form of listeriosis that makes up only a small percentage of all endocarditis cases [4].

Listeria IE presents with progressive symptoms of fever and general body illness, yet embolic events occur in 51% of cases, which exceeds typical IE infection rates. The high rate of systemic embolization in Listeria IE is attributed to the fragile, large vegetations that can form on valves and can shower septic emboli [4]. The mortality rate for Listeria IE becomes 37-50% when patients fail to receive prompt diagnosis and treatment. Even with therapy, one out of three patients may succumb to the infection [4].

Uniquely, L. monocytogenes has the propensity to cause simultaneous infections in multiple organ systems. While exceedingly rare, concurrent Listeria endocarditis and meningitis have been documented, especially in immunocompromised hosts, and are associated with poor outcomes [5].

Multiple documented cases demonstrate that Listeria infections can involve the CNS and endocardium, producing extensive disease spread and complicated diagnostic challenges. Reports show that early detection is crucial because standard treatment protocols for meningitis and endocarditis do not provide sufficient protection against Listeria infections [5,6].

Since L. monocytogenes is intrinsically resistant to cephalosporins, including ceftriaxone, CNS or cardiac infections due to Listeria will not respond to the usual third-generation cephalosporin-based empiric therapy for meningitis or community endocarditis [7]. The cornerstone of treatment is high-dose beta-lactam antibiotics (typically ampicillin or penicillin), often combined with an aminoglycoside for synergy [8].

We report a case of L. monocytogenes IE occurring simultaneously with bacterial meningitis and widespread septic embolization. We also review the relevant literature, highlighting the importance of recognizing this uncommon presentation and discussing optimal management strategies.

## Case presentation

A 74-year-old man with a history of benign prostatic hyperplasia (BPH) presented to the emergency department with 48 hours of high fever, progressive confusion, and decreased level of consciousness. He had no recent history of trauma, seizures, or focal neurological deficits before admission. On examination, his temperature was 39°Celsius, and he appeared disoriented, with a Glasgow Coma Scale score of 10, including eye opening to verbal stimuli (score 3), incomprehensible verbal responses (score 2), and localizing motor responses to pain (score 5), indicating a moderate reduction in the level of consciousness. Neurological examination revealed no signs of focal cranial nerve deficits or limb weakness, and reflexes were symmetric; thus, no gross focal neurological lesion was evident on exam. Cardiovascular exam initially noted a regular rhythm without murmurs. Other system exams were unremarkable except for the patient’s altered mental status.

Initial laboratory investigations revealed leukocytosis with a white blood cell count of 15,800 cells per microliter (normal range 4,000-10,000/µL), elevated C-reactive protein at 185 mg/L (normal <10 mg/L), and procalcitonin of 12 ng/mL (normal <0.5 ng/mL), consistent with a severe bacterial infection. Given the fever and confusion, an urgent septic workup was undertaken. Three sets of blood cultures were drawn, and a lumbar puncture was performed promptly due to suspicion of meningitis. Cerebrospinal fluid analysis showed 680 white blood cells per microliter, predominantly neutrophils (90%), with glucose measured at 28 mg/dL (normal range 45-80 mg/dL) and protein elevated to 185 mg/dL (normal range 15-45 mg/dL), findings consistent with acute bacterial meningitis. Gram stain of the CSF showed small gram-positive rods. Empiric broad-spectrum antimicrobial therapy was initiated immediately with intravenous ceftriaxone 2 grams every 12 hours and vancomycin 1,200 mg (15 mg/kg for a body weight of 82 kg) every 12 hours to cover common meningeal pathogens and possible endocarditis. Dexamethasone was administered adjunctively for suspected bacterial meningitis at a dose of 10 mg intravenously every six hours.

**Figure 1:**
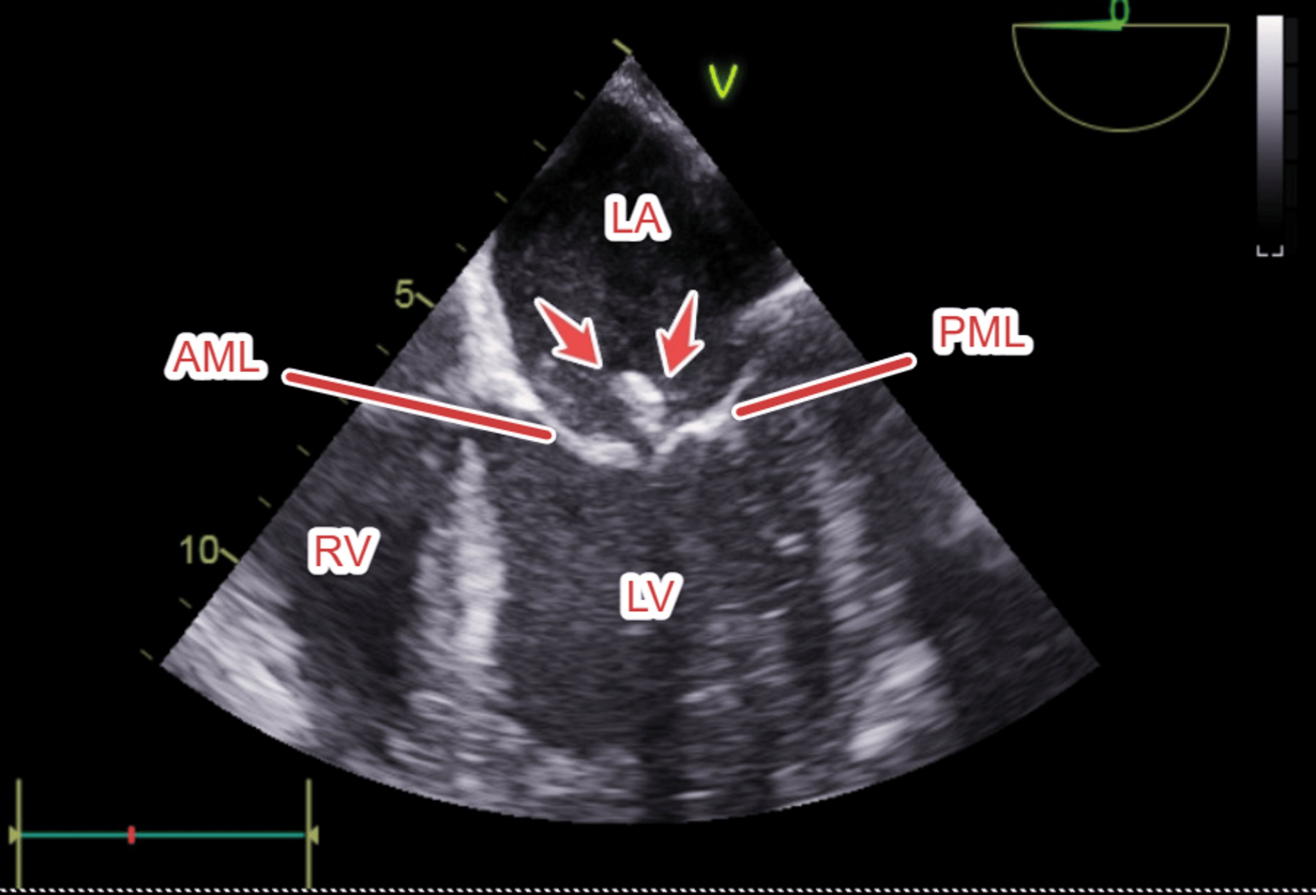
Mid-esophageal four-chamber TEE view at 0°, showing a mobile echogenic mass (red arrow) attached to the PML, consistent with mitral valve vegetation LA, left atrium; LV, left ventricle; RV, right ventricle; AML, anterior mitral leaflet; PML, posterior mitral leaflet; TEE, transesophageal echocardiography

During the early hospital course, the patient’s mental status remained depressed. Transthoracic echocardiography (TTE) was performed, given persistent fever, and showed a suspicious echodensity on the mitral valve, but had a poor acoustic window. Therefore, a transesophageal echocardiogram (TEE) was done and confirmed a 15x7 mm oscillating vegetation on the posterior leaflet of the mitral valve (Figure 1), establishing the diagnosis of IE. No obvious valve abscess or perforation was seen. This finding of IE provided a unifying explanation for the patient’s symptoms.

**Figure 2:**
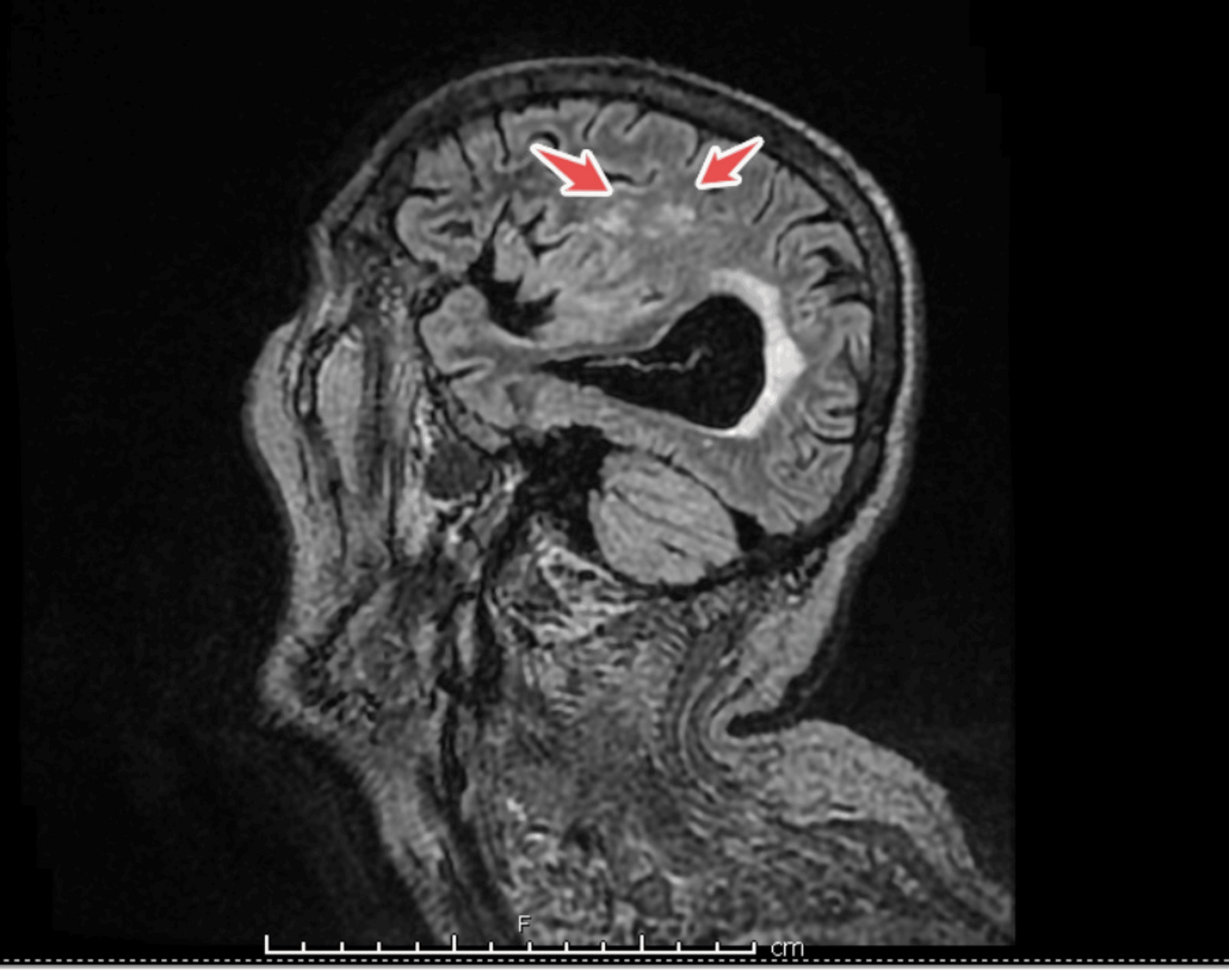
Sagittal MRI of the brain showing hyperintense lesions (red arrows), consistent with septic embolic lesions secondary to infective endocarditis MRI, magnetic resonance imaging

Magnetic resonance imaging (MRI) of the brain with diffusion-weighted sequences revealed multiple areas of acute infarction: a 9 mm acute infarct in the left cerebellum with diffusion restriction and fluid-attenuated inversion recovery (FLAIR) hyperintensity, and a small punctate infarct in the right frontal periventricular white matter. These lesions appeared embolic in nature. There were also chronic white matter ischemic changes appropriate for age. Magnetic resonance angiography (MRA) of the cerebral vessels showed no large artery occlusions or aneurysms. These neuroimaging findings corroborated the presence of multiple septic embolic strokes in the brain (Figure 2), likely originating from the infected cardiac valve.

**Figure 3:**
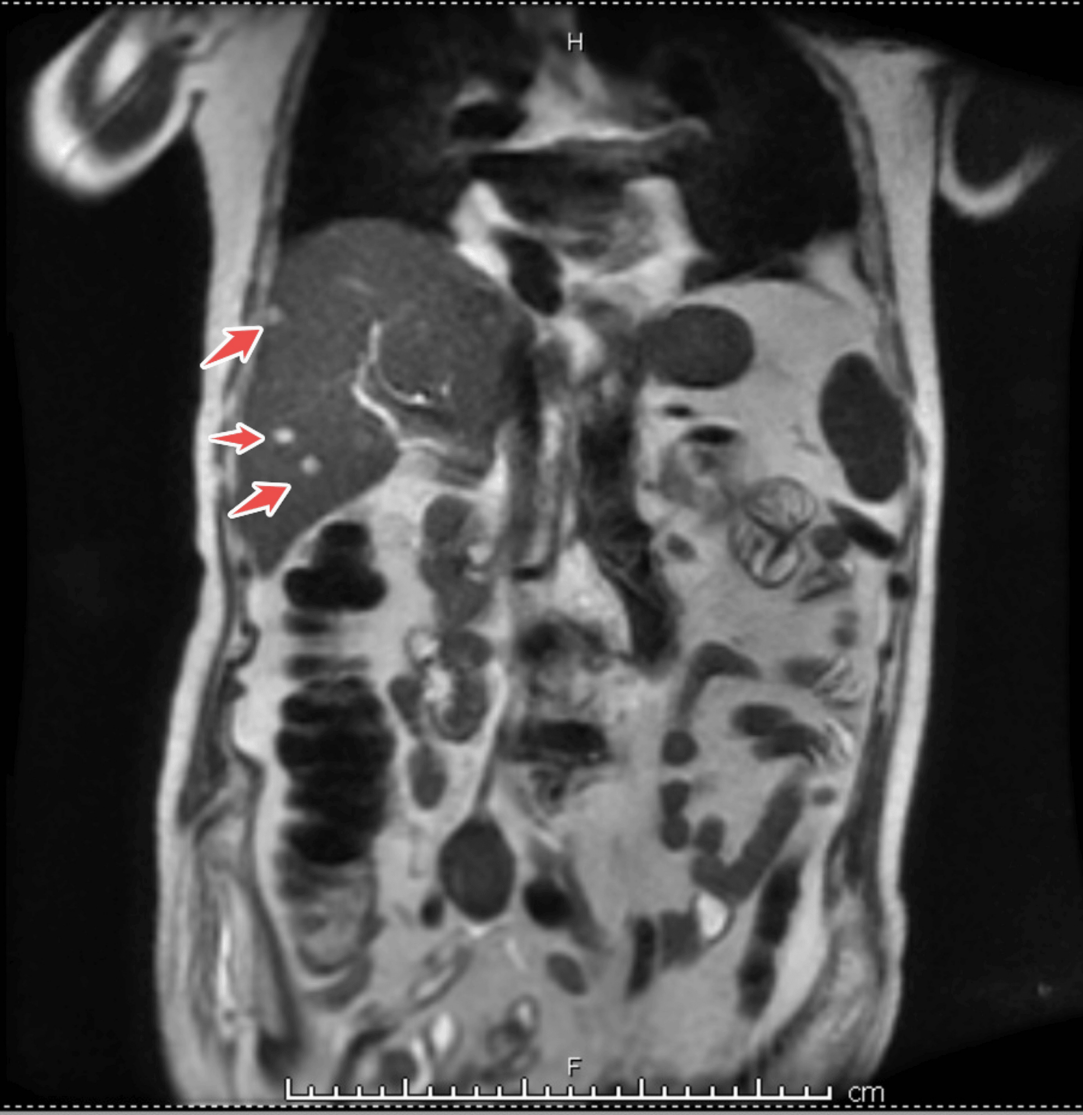
Coronal abdominal MRI demonstrates multiple focal hepatic lesions (highlighted by arrows) MRI, magnetic resonance imaging

**Figure 4:**
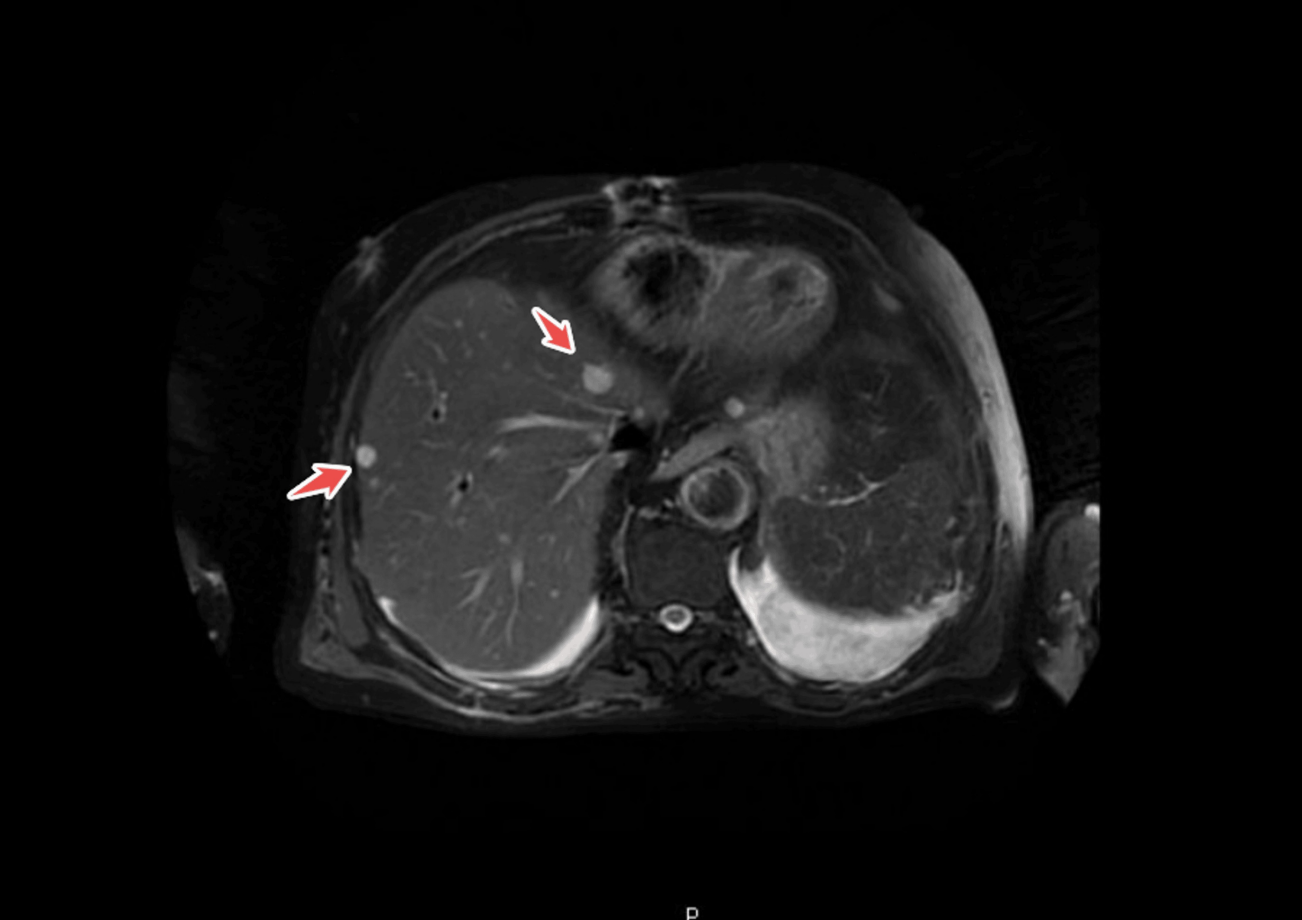
Axial abdominal MRI demonstrates multiple focal hepatic lesions (highlighted by arrows) MRI, magnetic resonance imaging

To evaluate for other systemic emboli, an abdominal contrast-enhanced CT scan was obtained. It demonstrated a wedge-shaped area of hypodensity in the spleen consistent with a splenic infarct, suggesting splenic embolization. Additionally, an infrarenal abdominal aortic aneurysm (AAA) measuring 4.7 cm in diameter with mural thrombus was incidentally noted, along with aneurysmal dilatation of the iliac arteries. There were small bilateral pleural effusions as well. Given the suspicion of septic emboli to solid organs, an abdominal MRI confirmed the splenic infarction and further revealed approximately 15 small hepatic lesions scattered in both liver lobes, up to 14 mm in size, with peripheral rim enhancement. These liver lesions were interpreted as microabscesses with a small septic embolic lesion in the liver (Figure 3 and Figure 4). No kidney infarcts or other abscesses were noted. The constellation of findings (brain infarcts, splenic infarct, and hepatic microabscesses) indicated widespread systemic septic embolization from the infected mitral valve.

Microbiological results became available within 48 hours. All three initial blood culture sets grew L. monocytogenes, confirming Listeria bacteremia associated with endocarditis. The CSF culture also grew L. monocytogenes, confirming that the meningitis was due to the same organism. Thus, the patient was diagnosed with L. monocytogenes IE, complicated by bacterial meningitis and multi-organ septic emboli. Antibiotic therapy was adjusted according to the culture results and known susceptibilities of Listeria. Ceftriaxone and vancomycin were discontinued (since Listeria is intrinsically resistant to cephalosporins and not covered by vancomycin), and the patient was started on high-dose intravenous ampicillin 2 grams every four hours in combination with gentamicin 245 mg per day (3 mg/kg/day for a body weight of 82 kg), administered in three divided doses of approximately 80 mg every eight hours to achieve synergistic bactericidal activity. This regimen is the recommended therapy for Listeria infections involving the endocardium or CNS, capitalizing on the bactericidal synergy between ampicillin and gentamicin.

The patient’s clinical course gradually improved on the targeted antibiotic therapy. His fever resolved within a week of therapy, and his mental status slowly cleared over the course of 10 days. No new neurologic deficits emerged. Renal function was monitored closely due to the gentamicin, which remained stable. By the end of three weeks of IV ampicillin with gentamicin therapy, the patient’s cognitive function had returned to near baseline, and he was ambulating with assistance. A repeat TEE at three weeks showed a reduction in vegetation size to 9 mm with no new lesions. Given the significant improvement, the decision was made to complete the antibiotic course at home. The patient was discharged to continue intravenous ampicillin via an outpatient parenteral antibiotic therapy (OPAT) program for a total of six weeks of therapy (with gentamicin given for the first three weeks). At a follow-up visit after completing antibiotics, he was doing well, neurologically intact except for some mild residual confusion, and with no signs of active infection.

## Discussion

This case highlights an unusual convergence of two serious infections, L. monocytogenes endocarditis and meningitis, along with systemic septic embolization to multiple organs. L. monocytogenes is an uncommon cause of IE. It accounts for a very small proportion of IE compared to typical pathogens like Staphylococcus, Streptococcus, or Enterococcus [9]. Patients with Listeria IE are often older adults with comorbidities or immunosuppressive conditions and frequently have underlying valvular abnormalities or prosthetic valves predisposing to infection [4]. Our patient fits the profile of an elderly male, though he had no known prosthetic valve or history of valvular disease. It is possible that minor age-related degenerative changes on the mitral valve or transient endothelial injury allowed Listeria to adhere and establish infection on the valve.

The main characteristic of Listeria endocarditis is its high rate of embolic events; vegetations in Listeria IE tend to be large and friable, showering infected emboli. Embolic events occur in approximately 50-60% of these patients [6].

Consistently, our patient had evidence of multi-organ septic embolization with multiple cerebral infarcts, a splenic infarct, and numerous microabscesses in the liver. Splenic infarcts are relatively common in endocarditis; more unusual in this case was the finding of multiple hepatic microabscesses due to Listeria. Hepatic involvement in listeriosis is rare, but it may present as solitary liver abscesses or, less commonly, multiple abscesses [10,11]. Reported cases of multiple liver abscesses caused by Listeria carried an extremely poor prognosis, with nearly all such patients dying despite treatment [11]. In contrast, our patient survived with appropriate antibiotic therapy. This favorable outcome may be due to early detection of the abscesses using MRI and the prompt initiation of effective antibiotics before the abscesses became too numerous or large. It also highlights improvements in supportive care and diagnostics since older case series. We did not pursue invasive drainage of the liver lesions, given their small size and expected response to antibiotics. By the end of therapy, the patient’s liver enzymes remained normal, and no signs of abscess complications were seen.

Another remarkable aspect of this case is the concurrent Listeria meningitis in the setting of endocarditis. L. monocytogenes is well known to cause meningitis, especially in neonates and the elderly, but the simultaneous occurrence of meningitis with endocarditis is uncommon [5]. In most cases of bacterial endocarditis, neurologic involvement is usually due to septic emboli causing strokes or brain abscesses rather than actual meningitis. Listeria, however, has a unique tropism for the CNS, often termed neurolisteriosis when it causes meningitis or encephalitis. There have been a few documented instances of combined Listeria endocarditis and meningitis [5]. In a recent case, Listeria endocarditis with meningoencephalitis (and even osteomyelitis by a secondary pathogen) was successfully treated in an immunosuppressed patient [6]. Our case adds to this literature as an example in which Listeria simultaneously caused endocarditis and meningitis in an elderly patient, in which the mechanism likely involved initial Listeria bacteremia from a foodborne source seeding both the cardiac valve and the meninges. The presence of embolic brain infarcts in our patient suggests that septic emboli from the valve may have also contributed to blood-brain barrier breakdown or direct seeding of bacteria into the CSF. Clinicians should be alert to this possibility, especially when a patient with endocarditis shows altered mental status or meningeal signs; concomitant meningitis should be considered, especially if the blood culture is growing Listeria or other organisms known to invade the CNS. Conversely, in an elderly patient with Listeria meningitis, assessment for endocarditis with echocardiography may be warranted if there are any suggestive signs, because finding and treating an unrecognized cardiac source can be lifesaving [5].

Management of Listeria endocarditis depends on timely, appropriate antibiotic therapy. Empiric regimens for endocarditis or meningitis often include ceftriaxone, but as mentioned, Listeria is intrinsically resistant to all cephalosporins. In retrospect, our patient’s empiric therapy of ceftriaxone and vancomycin did not cover Listeria (vancomycin has no activity against Listeria either). Our medical team identified the pathogen promptly, which allowed us to start specific treatment. The recommended treatment for Listeria infections that involve endocarditis and meningitis requires patients to receive high-dose intravenous ampicillin or penicillin G together with gentamicin as an aminoglycoside [10-12]. Ampicillin targets bacterial cell walls, while gentamicin works synergistically to create a bactericidal effect that speeds elimination of the pathogen from blood and cerebrospinal fluid [13].

Our patient received this regimen for a total of six weeks (with gentamicin given for three weeks). The recommended duration of gentamicin treatment remains uncertain because of its potential nephrotoxicity, yet a two to three week treatment period is commonly used for endocarditis when kidney function remains normal [14]. Penicillin-allergic patients should receive trimethoprim-sulfamethoxazole or meropenem because these medications are effective against Listeria [15]. The medical literature describes linezolid as a possible treatment for severe Listeria infections when beta-lactams fail, but beta-lactams remain the mainstay because they have proven effectiveness [16]. Our patient received ampicillin/gentamicin without complications, and we did not need to perform surgery because there were no indications of valve failure, abscess formation, or uncontrolled infection. The medical literature shows that cardiac surgery became necessary for only a quarter of Listeria IE cases because of either prosthetic valve involvement or refractory heart failure [17,18]. Our patient received effective treatment through medical therapy because his mitral regurgitation was not severe, and the vegetation size decreased with antibiotic therapy.

The patient received OPAT to complete his extended antibiotic treatment after becoming stable. This case demonstrates that proper coordination enables patients to receive treatment for severe Listeria endocarditis outside the hospital while maintaining safety standards, enhancing patient comfort, and shortening hospital stays. We ensured the patient had a peripherally inserted central catheter (PICC) and visiting nurse support; his family was educated on signs of relapse or complications. Follow-up blood cultures were negative, and imaging (repeat TEE) after therapy showed no residual infection.

From a public health perspective, an attempt was made to trace the source of the Listeria in this case. The patient’s family reported that he consumed unpasteurized dairy products, which are known to be high risk for listeriosis. It is likely that foodborne ingestion led to transient gut translocation of Listeria into the bloodstream. Given the patient’s advanced age and comorbidities, his immune system may have been less effective at clearing the bacteremia, allowing seeding of the cardiac valve and meninges. This underscores the importance of dietary counseling in high-risk individuals to avoid Listeria-prone foods.

## Conclusions

L. monocytogenes is a rare cause of endocarditis that can present concurrently with meningitis and systemic septic emboli, particularly in vulnerable individuals. This case highlights the importance of considering Listeria as a differential diagnosis of endocarditis with CNS involvement. Clinicians should be aware that L. monocytogenes is intrinsically resistant to cephalosporins, so standard empiric regimens need to be adjusted when this infection is suspected. The spread of Listeria IE emboli can reach multiple organs, including the brain, spleen, liver, and others, which requires complete evaluation for distant infection sites. Despite its severity, prompt diagnosis and a prolonged targeted antibiotic course can lead to full recovery even in cases with multi-organ involvement. This case adds to the growing evidence that combined Listeria endocarditis and meningitis, while uncommon, is a treatable condition when recognized early and managed aggressively. We emphasize preventive measures in high-risk populations, such as avoiding high-risk foods, and maintaining a high index of suspicion in appropriate clinical scenarios to improve outcomes.
